# Supplementary material for: Vitamin D Insufficiency Reduces Grip Strength, Grip Endurance and Increases Frailty in Aged C57Bl/6J Mice
Source: Nutrients. 2020 Sep 30;12(10):3005. doi: 10.3390/nu12103005 (PMC7599884; doi:10.3390/nu12103005)
Supplement: Supplementary file 1 [file nutrients-12-03005-s001.pdf]

Figure S1.

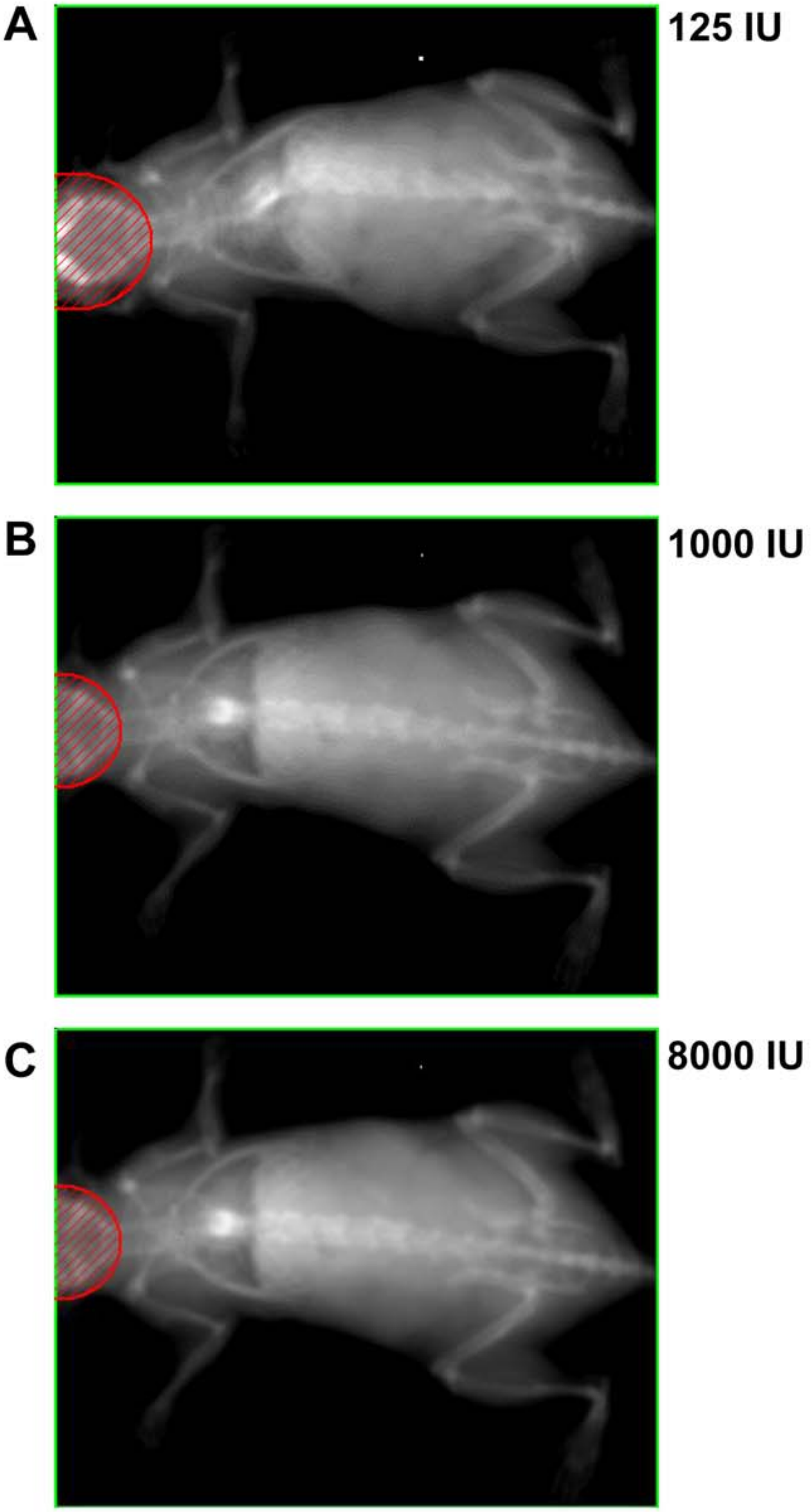

Supplemental Figure 1. Dual X-ray absorptiometry was used to acquire X-ray images of mice before and after 4 months of differential vitamin D supplementation. Representative images of mice closest to the group mean for bone mineral density at endpoint for 125IU (A), 1000IU (B), and 8000IU (C) supplementation groups, respectively.
